# BSA-Coated Metal–Phenolic Complex Assembly of 8-Shogaol Nanoparticles: Characterization, Stability, and Slow-Release Properties

**DOI:** 10.3390/foods15081365

**Published:** 2026-04-14

**Authors:** Rui Zhang, Xiao-Mei Ma, Kiran Thakur, Fei Hu, Jian-Guo Zhang, Yi-Long Ma, Zhao-Jun Wei

**Affiliations:** 1 School of Food and Biological Engineering, Hefei University of Technology, Hefei 230601, Chinahufei@hfut.edu.cn (F.H.); zhangjianguo@hfut.edu.cn (J.-G.Z.); yilong.ma@hfut.edu.cn (Y.-L.M.); 2 Ningxia Key Laboratory of Development and Utilization of Specialty Food Resources, School of Biological Science and Engineering, North Minzu University, Yinchuan 750021, China; 2025910@nmu.edu.cn

**Keywords:** 8-Shogaol, BSA, metal–phenolic network, nanoparticles

## Abstract

This study reports a self-assembled ternary delivery system composed of bovine serum albumin (BSA), Fe^(III)^, and 8-Shogaol (BSA-Fe^(III)^-8S) to enhance the stability of this labile ginger-derived bioactive compound. Optimized nanoparticles prepared via one-pot coprecipitation exhibited a particle size of 115.14 nm, polydispersity index (PDI) of 0.084, zeta potential of +52.23 mV, encapsulation efficiency of 94.93%, and loading capacity of 23.73%. Spectroscopic analyses (FT-IR, UV–Vis, XPS) and fluorescence quenching confirmed the formation of a core–shell metal–phenolic network, where Fe^(III)^ coordinates with 8-Shogaol and BSA forms the outer protein shell. Compared to free 8-Shogaol, the BSA-Fe^(III)^-8S MPN nanoparticles demonstrated significantly enhanced thermal, UV, and storage stability. During simulated gastrointestinal digestion, the nanoparticles retained 64.04% of 8-Shogaol, compared to only 51.38% for the free compound. Cytotoxicity assays on HEK293 cells confirmed the biocompatibility of the nanoparticles. This BSA-Fe^(III)^-8S delivery system offers a promising strategy for protecting bioactive phenolic compounds, with potential applications in functional foods and nutraceutical formulations.

## 1. Introduction

8-Shogaol, a major phenolic constituent of ginger (*Zingiber officinale* Roscoe), exhibits pronounced anti-inflammatory, antibacterial, and antitumor activities, rendering it a promising candidate for functional food and pharmaceutical development [1,2,3,4]. Our previous research has demonstrated that 8-Shogaol exerts significant inhibitory effects against colorectal cancer [5]. However, 8-Shogaol is highly labile and prone to rapid degradation under ambient light and thermal stress [6]. Therefore, it is imperative to develop strategies that can significantly enhance the stability and bioavailability of 8-Shogaol so that its therapeutic potential can be fully harnessed.

In recent years, self-assembly has emerged as a key focus in nano-encapsulation technology for enhancing nutrient stability, bioavailability, and sensory quality [7,8,9], with coordinate-based self-assembly offering a simple, universal approach to synthesize metal–phenolic network structures from diverse phenolic ligands and metal ions for various applications [8,10].

Fe^(III)^ plays a crucial role in nanoparticles due to their strong coordination ability, enabling robust binding to functional groups such as carboxyl and phosphate groups [11]. Consequently, through simple coprecipitation methods, trivalent iron ions can be utilized to directly coat the particle surface or subsequently modify functional molecules, thereby achieving targeted delivery. In contrast to most exogenous metals, Fe^(III)^ is endogenously required for oxygen transport and cellular energy metabolism; hence, ferric iron-based nanoparticles generally display favorable biocompatibility, undergo gradual physiological degradation, and are recycled via systemic iron homeostatic pathways, translating into minimal systemic toxicity. Polyphenolic compounds interact with various metal ions through hydrophobic interactions, hydrogen bonding, electrostatic forces, π–π stacking, and covalent bonds [7,12,13]. The properties of polyphenols have been extensively studied and summarized, with their strong metal chelation ability being a key characteristic. For instance, Ejima et al. [14] demonstrated that metal–polyphenol coordination polymers enable the coating of diverse materials. Consequently, while phenolic materials synthesized with polyphenols as primary building blocks are being explored in several emerging application fields, reports on Fe^(III)^ with 8-Shogaol to form nanoparticles remain scarce.

Accumulating evidence indicated that carriers fabricated from protein aggregates represents an effective and promising strategy for enhancing the aqueous dispersibility and physicochemical stability of bioactives under processing conditions [15,16]. For instance, corn zein nanoparticles effectively protect curcumin against photo-oxygen degradation and significantly enhance its absorption efficiency in the small intestine [17].

Bovine serum albumin (BSA), a natural, edible protein, serves as an ideal multifunctional biological template and stabilizer for constructing functional nanoscale delivery systems [18,19]. The amphiphilic nature of BSA enables it to stabilize oil–water interfaces, forming nanoemulsions or nanocomposites. Multiple binding sites within their molecular structure can stably interact with bioactive components through hydrophobic interactions and hydrogen bonds, providing protection. Hydrophobic regions on the BSA surface interact with the aromatic rings of polyphenols through hydrophobic interactions and π–π stacking. Extensive hydrogen bond networks form between the carbonyl and amino groups on BSA peptide chains and the numerous phenolic hydroxyl groups in polyphenols [20,21]. One study demonstrated that BSA/polyphenol complexes enhance the stability of high-internal-phase pickering emulsions [22]. Furthermore, Chen et al. [8] have demonstrated that BSA can successfully integrate with metal–phenolic network structures to exhibit specific biological functions. However, the interactions between BSA and Fe^(III)^-8S phenol–formaldehyde network nanoparticles remain undisclosed. We hypothesize that BSA interacts with Fe^(III)^-8S to construct novel carriers for bioactive substances and protect their physicochemical stability, which is a concept warranting further investigation.

Therefore, in this study, we developed a simple and universal method for the rapid synthesis of BSA-Fe^(III)^-8S nanoparticle system to enhance the stability and bioavailability of 8-Shogaol. We prepared and optimized BSA-Fe^(III)^-8S composite nanoparticles. This study evaluated multiple characteristics of BSA-Fe^(III)^-8S NPs, including particle size, zeta potential, encapsulation/drug loading efficiency, microstructure, and stability. We also measured the retention rate of BSA-Fe^(III)^-8S NPs during in vitro gastrointestinal digestion. Additionally, the cytotoxicity of BSA-Fe^(III)^-8S NPs was investigated using HEK293 cells. This study provides a novel ternary nanoparticle system, offering a new theoretical foundation for nutraceutical delivery systems.

## 2. Materials and Methods

### 2.1. Materials

8-Shogaol (purity 99%, Must Biotechnology Co., Ltd., Chengdu, China), whose chemical structure is shown in Figure S1. BSA (purity 98%, Yuanye Biological Technology Company, Shanghai, China), FeCl_3_ (Sinopharm Chemical Reagent Co., Ltd., Shanghai, China), fetal bovine serum (Thermo Fisher Scientific, Waltham, MA, USA). All the chemicals used in this study were of analytical grade.

### 2.2. Preparation of Nanoparticles

The preparation of nanoparticles via the single-pot assembly was based on the method of Chen et al. [6] with modifications. 8-Shogaol (200 μL, 10 mg mL^−1^), BSA (100 μL, 10 mg mL^−1^), and FeCl_3_ (200 μL, 4, 8, 16, 40, 80 mg mL^−1^) were successively added to a vial containing Milli-Q water (2 mL). BSA-Fe^(III)^-8S nanoparticles were prepared at 8-Shogaol: Fe^(III)^ molar ratios of 0.5:1, 1:1, 2:1, 5:1, and 10:1. The nanoparticles were stabilized by stirring the mixture at room temperature for 20 min. Excess material was removed by high-speed centrifugation (8000 g, 10 min). The resulting BSA-Fe^(III)^-8S nanoparticles were finally dispersed in Milli-Q water for subsequent use [10].

### 2.3. Particle Size, Zeta Potential, and Polydispersity Index (PDI)

Particle size, zeta potential, and polydispersity index (PDI) of the PCS-NPs were examined using a Zetasizer Nano-ZSE instrument (Malvern Instrument Ltd., Worcestershire, UK).

### 2.4. Encapsulation Efficiency and Loading Capacity

According to a previously reported method [8,23], free 8-Shogaol was extracted by mixing BSA-8S, Fe^(III)^-8S and BSA-Fe^(III)^-8S NPs with ethanol at a 1:3 (*v*/*v*) ratio and sonicating for 10 min. The supernatant was collected by centrifugation at 15,000 rpm for 20 min, and its encapsulation efficiency and drug loading capacity were determined. The content of 8-Shogaol in the samples was determined using Liquid Chromatography–Quadrupole Orbitrap Mass Spectrometry System (Thermo Fisher Scientific, Waltham, MA, USA) (the constructed standard curve was: y = 2 × 109x − 326458, R^2^ = 0.9999). We calculated the encapsulation efficiency and drug loading efficiency using the following formula:

(1) $$\mathrm{Encapsulation}\;\mathrm{efficiency}\;(\%)\;=\;\frac{Actual\;8-Shogaol}{Theory\;8-Shogaol}\times 100$$

(2) $$\mathrm{Loading}\;\mathrm{capacity}\;(\%)=\frac{Weight\;8-Shogaol\;in\;NPs}{Weight\;NPs}\times 100$$

### 2.5. Transmission Electron Microscopy (TEM) Observation

Nanoparticle morphology was examined by TEM (JEM-1400Flash, JEOL, Tokyo, Japan) at 120 kV; diluted samples were air-dried on carbon-coated copper grids for 5 min before imaging.

### 2.6. Fourier Transform Infrared Spectroscopy (FTIR)

Fourier transform infrared spectroscopy (FTIR) (Thermo Fisher, Waltham, MA, USA) spectra of 8-Shogaol, BSA, FeCl_3_, BSA-8S, Fe^(III)^-8S and BSA-Fe^(III)^-8S were acquired from 4000 to 500 cm^−1^ to compare vibrational features.

### 2.7. Ultraviolet–Visible Spectra Determination

The UV–visible absorption spectra of 8-Shogaol and nanoparticle samples were recorded on a UV–visible spectrophotometer (Jasco, Tokyo, Japan), covering the absorbance wavelength range of 200 to 800 nm [23].

### 2.8. X-Ray Photoelectron Spectroscopy (XPS) Analysis

The elemental composition of BSA-8S, Fe^(III)^-8S, and BSA-Fe^(III)^-8S were analyzed using X-ray photoelectron spectroscopy (Thermo Fisher Scientific, Waltham, MA, USA).

### 2.9. Atomic Force Microscopy (AFM) Analysis

The microstructures of samples (8-Shogaol, BSA, FeCl3, BSA-8S, Fe^(III)^-8S and BSA-Fe^(III)^-8S NPs) were observed using a Dimension Icon microscope (Bruker Corporation, Billerica, MA, USA). Samples were pipetted onto freshly cleaved mica sheets. After air drying, atomic force microscopy images were acquired and analyzed using Nano Scope analysis software (Version 1.5).

### 2.10. Thermal Gravitational Analysis (TGA)

The weight loss of 8-Shogaol, BSA-8S, Fe^(III)^-8S, and BSA-Fe^(III)^-8S NPs nanoparticles was measured using a thermogravimetric analyzer (NETZSCH, Bavaria, Germany) under 25–600 °C in nitrogen atmosphere at a heating rate of 10 °C/min. Differential thermal gravimetric (DTG) analysis was then performed, which is the derivative of the TG curve with respect to temperature.

### 2.11. Fluorescence Quenching Analysis

Fluorescence scans of BSA, BSA-8S, BSA-Fe^(III)^ and BSA-Fe^(III)^-8S samples were performed using a fluorescence spectrometer (F98, Shanghai Linguang Technology Co., Ltd., Shanghai, China) [24]. The samples were scanned for their spectral profiles in the 290–450 nm range at a fixed excitation wavelength of 280 nm.

To analyze the fluorescence quenching mechanism of the samples, we performed further calculations on the fluorescence intensity data using the Stern–Wolmer equation and the double-logarithmic equation:

(3) F0/F=1+Ksv[Q]=1+Kqτ0[Q]

(4) log[(F0−F)/F]=logKa+nlog[Q]

where F_0_ and F represent the fluorescence intensities of the protein molecules (BSA, BSA-Fe^(III)^ and the protein molecules containing 8-Shogaol, respectively. Ksv and Kq denote the quenching constant and quenching rate constant, respectively. [Q] is the concentration of 8-Shogaol. τ_0_ is the fluorophore lifetime. Ka and n represent the binding constant and the number of binding sites, respectively.

### 2.12. Thermal Stability

To investigate the thermal stability of 8-Shogaol, BSA-8S, Fe^(III)^-8S, and BSA-Fe^(III)^-8S, samples were heated at 30, 40, 50, 60, 70, 80, and 90 °C for 1 h, after which 200 μL aliquots were removed for quantitative analysis. Absorbance at 288 nm was measured, and the 8-Shogaol concentration was calculated using the standard curve equation established in this study (y = 4.3257x + 0.0276, R^2^ = 0.998).

### 2.13. UV Irradiation Stability

To evaluate the stability of BSA-8S, Fe^(III)^-8S and BSA-Fe^(III)^-8S under ultraviolet irradiation of different durations, the nanoparticles and 8-Shogaol monomers were co-placed in transparent glass test tubes and subjected to ultraviolet light exposure for 0, 2, 4, 6, 8, 10 and 12 h, respectively. The retention rate of 8-Shogaol was subsequently determined.

### 2.14. Storage Stability

The retention rate of 8-Shogaol and nanoparticles were also measured after storage at 4 °C for 0, 5, 10, 15, 20, 25, and 30 days to investigate their storage stability.

### 2.15. In Vitro Digestion Simulation

The simulated digestion experiment for 8-Shogaol BSA-8S, Fe^(III)^-8S, and BSA-Fe^(III)^-8S nanoparticles were conducted using the method described by Yang et al. [24]. Briefly, 10 mL of the sample was mixed with simulated gastric fluid (20 mL), composed of 0.1 M hydrochloric acid, 3.2 mg/mL pepsin, and 0.2 mg/mL sodium chloride (pH 2). After shaking at 37 °C and 100 rpm for 2 h, the mixture was adjusted to pH 7.0 to terminate simulated gastric digestion. Subsequently, 10 mL of the digested solution was combined with an equal volume of simulated intestinal fluid containing 2 mg/mL trypsin, 10 mg/mL bile salts, and 6.8 mg/mL potassium dihydrogen phosphate. The mixture was incubated under the same conditions for 2 h. During the in vitro digestion process, digestion solutions of the 8-Shogaol, BSA-8S, Fe^(III)^-8S, and BSA-Fe^(III)^-8S complexes were collected every 30 min to determine the retention rates of 8-Shogaol.

(5) $$\mathrm{Retention}\;\mathrm{rate}\;(\%)=\frac{M1}{M2}$$

where M1 is the remaining amount of 8-Shogaol and M2 is the initial total amount of 8-Shogaol.

### 2.16. Cytotoxicity Evaluation

MTT assay (Beijing Solarbio Science & Technology Co., Ltd., Beijing, China) was conducted to evaluate the cytotoxicity of 8-Shogaol BSA-8S, Fe^(III)^-8S, and BSA-Fe^(III)^-8S nanoparticles on HEK293 cells (Wuhan Procell Life Science & Technology Co., Ltd., Wuhan, China). Cells were seeded at a density of 1 × 10^5^ cells/well (100 μL) in a 96-well plate. The culture medium used for HEK293 cells was high-glucose DMEM (containing 10% fetal bovine serum), purchased from Gibco (Thermo Fisher Scientific, Waltham, MA, USA). After 24 h, nanoparticle solutions and free 8-Shogaol solutions at different concentrations were supplemented into the culture medium, and the cells were further incubated at 37 °C for 24 h. The medium was then discarded, and 120 μL of MTT solution (5 mg/mL) was added; the mixture was incubated for another 4 h. Subsequently, 150 μL of dimethyl sulfoxide (DMSO) was introduced into the system. Following 10 min of shaking, the absorbance value at a wavelength of 490 nm was determined using a spectrophotometer [23].

### 2.17. Statistical Analysis

All experiments were independently repeated at least three times, and the results are expressed as mean ± standard deviation (mean ± SD). Statistical analysis was performed using SPSS 26.0 software. Comparisons among multiple groups were conducted using one-way analysis of variance (ANOVA), followed by Tukey’s HSD post hoc test for multiple comparisons. A *p* value < 0.05 was considered statistically significant.

## 3. Results and Discussion

### 3.1. Particle Size, Zeta Potential, PDI, EE and LC of Samples

**Figure 1:**
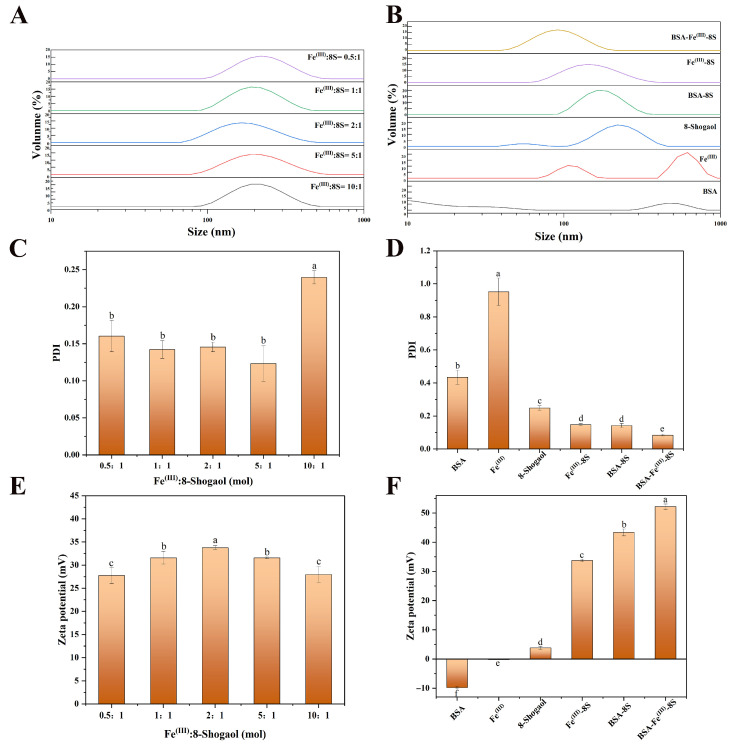
The particle size distribution curves of the samples include (**A**) Fe^(III)^:8S at different molar ratios, as well as (**B**) BSA, Fe^(III)^, 8-Shogaol, BSA, BSA-8S, Fe^(III)^-8S, and BSA-Fe^(III)^-8S NPs; (**C**) the PDI of Fe^(III)^:8S at different molar ratios, as well as BSA, Fe^(III)^, 8S, BSA, BSA-8S, Fe^(III)^-8S, and (**D**) BSA-Fe^(III)^-8S NPs; (**E**) zeta potential of Fe^(III)^:8S at different molar ratios, as well as (**F**) BSA, Fe^(III)^, 8S, BSA, BSA-8S, Fe^(III)^-8S, and BSA-Fe^(III)^-8S NPs. Data are expressed as mean ± standard deviation (*n* = 3) (*p* < 0.05). Different lowercase letters indicate significant differences among the groups (*p* < 0.05).

The particle size, polydispersity index (PDI), and zeta potential of Fe^(III)^-8S nanoparticles with different molar ratios were determined using dynamic light scattering. As shown in Figure 1A, the particle size distribution curves for Fe^(III)^:8S ratios of 0.5:1, 1:1, 2:1, 5:1, and 10:1 indicated that the smallest particle size of 166.13 nm was achieved at a Fe^(III)^:8S ratio of 2:1, indicating that Fe^(III)^ initially increased cross-linking density. Beyond this ratio, particle size enlarged and irregular aggregates appeared, revealing an optimal Fe^(III)^ loading above which excess iron destabilized the system. This was consistent with the findings of Chen et al. [8] which reported a minimal BSA/Fe/tannic acid particle size of 105 nm at a Fe: tannic acid molar ratio of 1:1, emphasizing that an optimal metal–phenolic stoichiometry was required to maximize cross-linking while avoiding iron-induced aggregation.

The corresponding zeta potential values were 27.77 mV, 31.60 mV, 33.77 mV, 31.57 mV, and 27.97 mV, respectively (Figure 1C). The PDI values were 0.16, 0.14, 0.12, 0.15, and 0.24, respectively (Figure 1E). Results indicated that at a molar ratio of 2:1, the absolute zeta potential value was the highest (33.77 mV) and the PDI was the lowest (0.12), demonstrating excellent stability and uniformity of nanoparticles synthesized at this concentration. Therefore, this concentration was selected for subsequent experiments.

Subsequently, the Fe^(III)^-8S nanoparticles were surface-coated with BSA to yield BSA-Fe^(III)^-8S nanoparticles. Compared to the three monomers BSA, 8-Shogaol, and Fe^(III)^, the particle size and PDI values of BSA-8S, Fe(III-8S, and BSA-Fe^(III)^-8S were significantly reduced, while the absolute values of zeta potential increased significantly (Figure 1B). BSA-Fe^(III)^-8S exhibited the optimal encapsulation effect (particle size of 115.13 nm, PDI of 0.084, and Zeta potential of 52.23 mV) (Figure 1D,F). The high absolute zeta potential value of +52.23 mV indicated strong electrostatic repulsion among the nanoparticles, which is a critical factor for maintaining colloidal stability and preventing aggregation [19,23]. The encapsulation efficiencies of BSA-8S, Fe^(III)^-8S, and BSA-Fe^(III)^-8S were 84.77%, 84.54%, and 94.93%, respectively, with drug loading capacities of 21.14%, 21.20%, and 23.73% (Table 1). The above results demonstrated that the BSA-Fe^(III)^-8S system markedly increased both the encapsulation efficiency and drug-loading capacity of 8-Shogaol.

Consistent with the findings of Ma et al. [23], the synthetic ZLC ternary nanosystem exhibited significantly higher encapsulation efficiency and loading capacity. When 8-Shogaol and Fe^(III)^ are directly mixed, they may randomly encounter each other in solution and rapidly cross-link, forming larger MPN aggregates. This results in an uneven particle size distribution, with larger particles predominating. In the presence of BSA, the surface of protein molecules is rich in functional groups such as amino and carboxyl groups. These groups can pre-bind iron through coordination interactions, anchoring these reactants within their three-dimensional structures. Upon addition of polyphenols, these compounds reacted exclusively with metal ions pre-fixed at specific sites on the protein [8]. This confined MPN formation to the protein surface or internal cavities, resulting in smaller, more uniform final particle sizes.

### 3.2. TEM Observation Analysis

**Figure 2:**
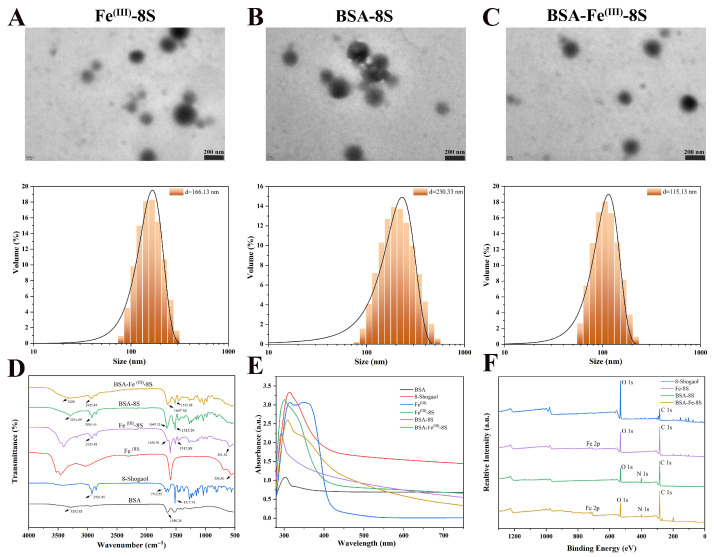
Characterization of different nanoparticle groups. TEM images and particle size distribution of (**A**) Fe^(III)^-8S, (**B**) BSA-8S, and (**C**) BSA-Fe^(III)^-8S NPs; (**D**) FTIR spectra and (**E**) UV–vis spectra of BSA, Fe^(III)^, 8-Shogaol, BSA-8S, Fe^(III)^-8S, and BSA-Fe^(III)^-8S NPs; (**F**) XPS survey analyses of 8-Shogaol, BSA-8S, Fe^(III)^-8S, and BSA-Fe^(III)^-8S NPs.

The morphology of Fe^(III)^-8S, BSA-8S, and BSA-Fe^(III)^-8S were characterized using TEM (Figure 2A–C). Fe^(III)^-8S nanoparticles display a spherical morphology; however, they exhibit considerable heterogeneity in both size and shape, with an average diameter of 166.13 nm, indicative of limited uniformity. BSA-8S nanoparticles likewise present a spherical morphology, confirming the successful encapsulation of 8-Shogaol by BSA. Nevertheless, pronounced aggregation yields larger entities, with a mean diameter of 230.33 nm. By contrast, BSA-Fe^(III)^-8S nanoparticles displayed the narrowest size distribution and the smallest mean diameter (115.13 nm). This may be attributed to the role of BSA as a structure-directing agent, whose amphiphilic surface establishes electrostatic and hydrophobic interactions with Fe^(III)^ and 8-Shogaol, thereby constructing a tighter cross-linked network while providing steric hindrance stability to suppress aggregation.

### 3.3. FTIR

FTIR was employed to investigate the structural characteristics and interaction relationships of 8-Shogaol, BSA, Fe^(III)^, BSA-8S, Fe^(III)^-8S, and BSA-Fe^(III)^-8S. In the BSA spectrum, the broad absorption band at 3300–3000 cm^−1^ is attributed to N-H stretching of the protein backbone. The peaks at 1650 cm^−1^ and 1540 cm^−1^ correspond to amide I (primarily C=O stretching) and amide II (N-H bending and C-N stretching), respectively. Additionally, the bands between 1400 and 1200 cm^−1^ arise from C-H bending and C-N stretching vibrations [25]. The broad absorption band at 3500–3200 cm^−1^ in 8-Shogaol originated from O-H stretching of phenolic hydroxyl groups. The peak at 2900 cm^−1^ corresponds to C-H stretching and asymmetric/symmetric stretching vibrations of –CH_2_– groups in long alkyl side chains. The 1700–1720 cm^−1^ range corresponds to C=O stretching, while the peaks at 1600 and 1510 cm^−1^ represent C=C stretching in aromatic rings and alkenes [26,27]. Multiple sharp dips at 1250–1150 cm^−1^ result from the coupling of phenolic C-O stretching with in-plane O-H bending vibrations, forming characteristic peaks for phenolic compounds [8]. The spectrum of Fe^(III)^ primarily exhibited vibrations from water molecules and metal–halide bonds. Characteristic peaks include a minor broad absorption at 3400 cm^−1^ (likely O-H from hydration or impurities in the salt). Anhydrous ferric chloride is highly hygroscopic, readily absorbing moisture from the air to form hydrated iron ions. The strong peak at 1620 cm^−1^ corresponds to H-O-H bending vibration (water molecular deformation mode). The 600–500 cm^−1^ range may correspond to Fe-O stretching vibrations [28].

FT-IR confirmed formation of Fe^(III)^-8S nanoparticles: the fingerprint features of 8-Shogaol are preserved, yet the C=O stretch moves from 1710 cm^−1^ to 1650 cm^−1^, indicating keto-oxygen coordination to Fe^(III)^ and consequent bond weakening. A new weak band at 591 cm^−1^ was assigned to (Fe-O), verifying that the interaction was chemical rather than a simple physical blend. BSA-8S nanoparticles exhibited the amide I (1650 cm^−1^) and amide II (1540 cm^−1^) signatures of BSA alongside the retained aromatic/alkene bands of 8-Shogaol (1600 and 1510 cm^−1^) and its rippled fingerprint. The 8-Shogaol C=O stretch merged into and broadened the amide I envelope (shift from 1700 cm^−1^ to 1650 cm^−1^), indicating attachment via hydrogen bonding, covalent linkage or encapsulation. Enhanced signals at 1250–1150 cm^−1^ integrated the C-O features of 8-Shogaol. Unchanged amide positions with only minor intensity variations demonstrated that the protein secondary structure remains intact during 8-Shogaol interaction, confirming successful conjugation without degradation.

FT-IR spectra of the BSA-Fe^(III)^-8S nanoparticles exhibited combined signatures of all three components: amide I/II bands of BSA at 1650 and 1550 cm^−1^, the fingerprint undulation of 8-Shogaol between 1500 and 500 cm^−1^, and weak Fe^(III)^ features at low wavenumbers. The C=O stretching band of 8-Shogaol (1710 cm^−1^) overlapped with amide I and shifted downward to 1647 cm^−1^, indicating coordination of phenolate oxygen to Fe^(III)^ and a consequent decrease in electron density of the conjugated system. The O-H stretching region (3500–3200 cm^−1^) broadened and exhibited moderate intensity, revealing partial disappearance of phenolic O-H of 8-Shogaol due to deprotonation, while the remaining signal originated mainly from N-H of BSA and trace water. The characteristic 1620 cm^−1^ H-O-H bending band of FeCl_3_ almost vanished, suggesting that Fe^(III)^ converted from a hydrated state to a phenolate–metal complex with partial release of water. A small peak emerged at 500–600 cm^−1^, attributable to Fe-O or Fe-phenolate vibrations which serve as a hallmark of the metal–phenolic network. Collectively, these spectroscopic signatures confirmed the successful construction of a metal–phenolic network wherein Fe^(III)^ acts as a central cross-linking node, coordinating with the phenolate oxygens of 8-Shogaol.

### 3.4. UV–Vis Spectroscopy

UV–vis spectroscopy was employed to probe the structural features and interactions among 8-Shogaol, BSA, Fe^(III)^, BSA-8S, Fe^(III)^-8S, and BSA-Fe^(III)^-8S. The characteristic absorption of 8-Shogaol at 313 nm exhibited a distinct blue shift in BSA-8S, Fe^(III)^-8S, and BSA-Fe^(III)^-8S, indicating that the aromatic chromophore of 8-Shogaol experienced a less polar and more constrained microenvironment, which raises the energy required for π→π transitions [29]. BSA displayed a strong absorption peak at approximately 300 nm, originating from the aromatic amino-acid residues (tryptophan and tyrosine). In BSA-8S, the blue shift was primarily attributed to the transfer of 8-Shogaol from the polar aqueous phase into the hydrophobic pocket of BSA, accompanied by disruption of the original hydrogen bond network. In Fe^(III)^-8S, absorption peaks emerged at 303 nm and 335 nm. This shift reflected the formation of Fe-phenolate coordination bonds, which perturb the electronic structure of 8-Shogaol, thereby providing spectroscopic evidence for the metal–polyphenol network structure. This observation was analogous to the findings reported by Tardy et al. [30], where UV–vis spectroscopic analysis demonstrated increased absorbance at approximately 335 nm for (TA/Fe^III^)_3_ films functionalized with P(EtOx)-Gal. When BSA, Fe^(III)^, and 8-Shogaol are combined to form nanoparticles, the blue shift in the maximum absorption peak accompanied by decreased absorbance results from the combined effects of multiple physicochemical processes. The blue shift in the maximum absorption peak primarily rose from fundamental alterations in the electronic structure and microenvironment of the chromophore (8-Shogaol). Fe coordination reduces electron density, while BSA provides hydrophobic encapsulation [28]. This phenomenon also confirmed the successful synthesis of the ternary composite nanoparticles through spectroscopic characterization.

### 3.5. XPS Analysis

The results of XPS analysis are shown in Figure 1F. XPS spectra were employed to evaluate the elemental composition on the carrier surface, providing chemical information about the outermost layer (1–10 nm) of the nanoparticles [31,32]. Compared to the 8-Shogaol monomer, Fe^(III)^-8S exhibited a distinct Fe 2p peak at 711 eV, confirming successful Fe incorporation. The C and O signal intensities showed slight decreases, indicating surface coverage by the Fe^(III)^-8S network. In BSA-8S, the Fe 2p peak disappeared while the N 1s signal significantly increased (BSA contains abundant amide nitrogen). The C and O signals remained stable. O signals slightly decreased, indicating surface coverage by the Fe^(III)^-8S network. In BSA-8S, the Fe 2p peak disappeared, the N 1s signal significantly enhanced (BSA contains abundant amide nitrogen), and the C and O peak shapes were essentially consistent with 8-Shogaol, suggesting a protein-dominated surface after BSA encapsulation.

In the BSA-Fe^(III)^-8S system, simultaneous detection of Fe 2p, N 1s, C 1s, and O 1s peaks were observed. The intensity of the Fe 2p peak was lower than that in Fe^(III)^-8S but higher than in BSA-8S (which showed zero intensity), indicating that Fe was successfully encapsulated within the BSA-8S composite layer. This formed a ternary core–shell structure, serving as the most direct evidence for the successful formation of the ternary composite. In BSA-Fe^(III)^-8S, the C 1s signal was the most intense peak, originating predominantly from the peptide backbone and amino-acid side chains of BSA, as well as from the aromatic rings and alkyl chains of 8-Shogaol. This signal served as the most prominent carbon signature and reference for the entire organic matrix. The O 1s peak originated from all oxygen-containing components, including the peptide bonds and carboxyl groups of BSA, the phenolic hydroxyls of 8-Shogaol, and the Fe-O coordination bonds that formed the metal–phenolic network. The N 1s signal served as a marker for BSA presence, since nitrogen was almost exclusively supplied by the peptide bonds and amino groups of the protein; its detection confirmed that BSA had been successfully incorporated into the nanoparticle surface. The Fe 2p peak, positioned at 711 eV with a satellite near 724 eV, provided unambiguous evidence for Fe^(III)^. This signal can be attributed to Fe^(III)^ in diverse coordination environments, indicating that Fe^(III)^ exists as mixed-ligand complexes coordinated with phenolic oxygen from 8-Shogaol and carboxyl/amino groups from BSA. Its intensity was attenuated relative to Fe^(III)^-8S, directly indicating that the iron was enveloped by an outer BSA shell, thereby forming a core–shell architecture rather than surface-exposed clusters and confirming successful construction of the metal–phenolic network. This result was consistent with the findings of Kou et al. [33], who developed metal–polyphenol networks encapsulated in sodium alginate microsphere hydrogels for the delivery of catechin and vitamin C. In the work of Lei et al. [34] the progressive rise in C/Si ratio together with the emergence of an N 1s peak for MSN-SS-PDA verified that each surface-modification step had been completed successfully.

### 3.6. AFM

**Figure 3:**
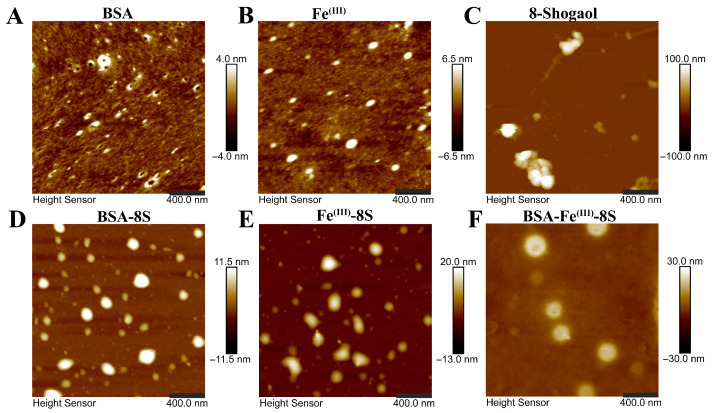
AFM images of (**A**) BSA, (**B**) Fe^(III)^, (**C**) 8-Shogaol, (**D**) BSA-8S, (**E**) Fe^(III)^-8S, and (**F**) BSA-Fe^(III)^-8S NPs.

The microscopic structure of the sample was observed using atomic force microscopy (AFM), with the image shown in Figure 3. BSA appeared as nano-sized, compact, round particles with a broad size distribution, a morphology consistent with that reported by Wu et al. [35]. Fe^(III)^ presented as ellipsoidal grains of non-uniform dimensions, while 8-Shogaol formed irregular aggregates. BSA-8S nanoparticles exhibited polydisperse, roughly spherical architectures that retained the characteristic features of BSA. Upon chelation between Fe^(III)^ and 8-Shogaol, the resultant Fe^(III)^-8S phenolic network nanoparticles exhibited irregular and polydisperse morphologies (Figure 3D), a finding analogous to that of Xu et al. [10], who reported similarly non-uniform, roughly spherical metal–phenolic nanoparticles prepared from Fe(II)-quercetin complexes. The BSA-Fe^(III)^-8S nanoparticles exhibited dense and monodisperse spherical morphologies, in agreement with earlier TEM observations and thus verifying the successful fabrication of the nanoparticles.

### 3.7. Fluorescence Quenching Analysis of Various Samples

**Figure 4:**
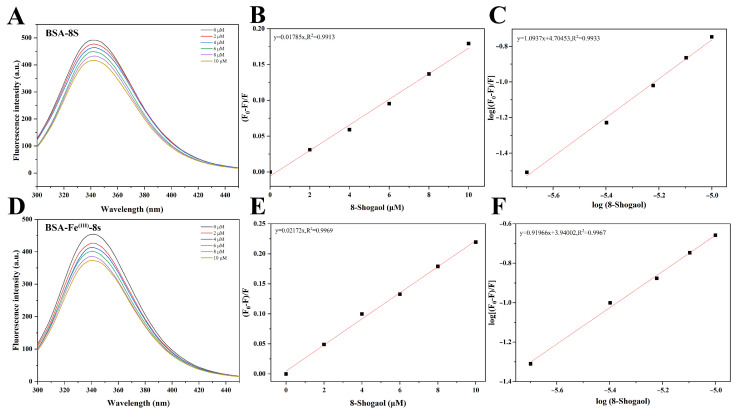
Emission fluorescence spectra of (**A**) BSA, and (**D**) BSA-Fe^(III)^ complexes with various concentrations of 8-Shogaol (0–10 μM). (**B**,**C**) The Stern–Volmer plots for quenching of the BSA; (**E**,**F**) BSA-Fe^(III)^ complexes by 8-Shogaol.

Protein–ligand interactions and their binding status can be revealed by intrinsic fluorescence emission spectroscopy. As illustrated in Figure 4, the emission intensities of BSA-8S and BSA-Fe^(III)^-8S were lower than that of native BSA, confirming the interaction between the protein and 8-Shogaol. When the concentration of 8-Shogaol was raised from 0 to 10 μM, the fluorescence of the BSA-8S nanoparticle complex decreased in a dose-dependent manner, indicating that 8-Shogaol quenches the protein’s intrinsic fluorescence in a concentration-dependent fashion. This indicated that intermolecular energy transfer occurred between the ligand and the tryptophan residues of the protein. The small molecule bound to the protein through non-covalent interactions, such as hydrophobic forces, electrostatic forces, and hydrogen bonding, resulting in the formation of a non-fluorescent complex [36]. The quenching mechanism involved in complexes formed between proteins and small molecules primarily refers to fluorescence quenching, which was the phenomenon where the intrinsic fluorescence of a protein (mainly originating from tryptophan and tyrosine residues) decreases after binding with a small molecule. Based on their mechanisms of action and kinetic characteristics, they can be classified into two types: static quenching and dynamic quenching. In the static quenching mode, the reduction in fluorescence intensity stems from the ground-state complexation between the quencher and the fluorescent substance, whereas dynamic quenching results from collisions between these molecules [37].

The linear Stern–Volmer plots for the samples are shown in Figure 4B,E, with the Ksv and Kq parameters listed in Table 2. All sample fits exhibited high correlation coefficients (R^2^ > 0.99). The results indicated that the Kq values of all samples were significantly higher than 2 × 10^10^ mol^−1^ LS^−1^ (the maximum diffusion-limited quenching constant), suggesting that the BSA-8S and BSA-Fe^(III)^-8S nanoparticle complexes conform to the static fluorescence quenching mechanism. Similarly, Lee et al. [38] reported that the interaction between serum albumin and propyl gallate and methyl gallate follows a static quenching model. As summarized in Table 2, the Ksv values for BSA-8S and BSA-Fe^(III)^-8S nanoparticle complexes were 1.7850 × 10^3^ mol^−1^ L and 2.1720 × 10^3^ mol^−1^ L, respectively, indicating stronger interaction forces between the BSA-Fe^(III)^ nanoparticle complex and 8-Shogaol. This enhancement may be attributed to Fe^(III)^ coordination inducing conformational changes in the protein structure or creating additional binding sites, thereby enhancing the accessibility of 8-Shogaol to the fluorophore. Figure 4C,F display the double-log regression curves of 8S-mediated fluorescence quenching for BSA and BSA-Fe^(III)^. As shown in Table 1, the binding site number between 8S molecules and the protein/binary nanoparticle complexes approached 1, indicating a single binding site between them. Notably, the Ka value of the BSA-Fe^(III)^-8S complex (8710 mol^−1^ L) was lower than that of the BSA-8S (50,644 mol^−1^ L), indicating higher binding affinity between BSA and 8-Shogaol. The lower Ka value may indicated that, following Fe^(III)^ coordination, the binding mode shifts from high-affinity specific binding to a more dynamic or multi-site interaction pattern; however, more effective fluorescence quenching still occurs due to the favorable spatial positioning of the quencher relative to the tryptophan residues. This interpretation aligns with the proposed model in which BSA serves as a structural directing agent, organizing the formation of Fe^(III)^-8S networks on or near the protein surface, thereby enhancing the stability and protective effect for the bioactive compound. A similar study by Roy et al. [39] revealed that quercetin-Cu^(II)^ complexes can quench the fluorescence of serum albumin, suggesting that metal ion complexation induces a shift in the binding mode from high-affinity specific binding toward dynamic/multi-site interactions.

### 3.8. Thermogravimetric Analysis

**Figure 5:**
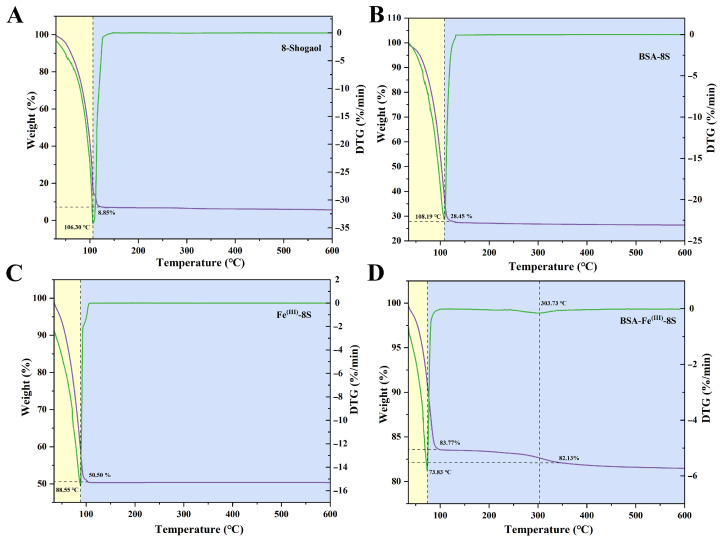
TGA and DTG analyses of (**A**) 8-Shogaol, (**B**) BSA-8S, (**C**) Fe^(III)^-8S, and (**D**) BSA-Fe^(III)^-8S NPs.

The results of thermogravimetric analysis revealed that the initial weight loss below 150 °C in all groups was primarily attributed to moisture evaporation [32]. The 8-Shogaol monomer group exhibited the highest weight loss rate, ultimately reaching 8.85%. The final weights for the BSA-8S, Fe^(III)^-8S, and BSA-Fe^(III)^-8S groups reached 28.45%, 50.50%, and 82.13%, respectively, showing significant improvements compared to the 8-Shogaol monomer, particularly in the BSA-Fe^(III)^-8S group. The BSA-Fe^(III)^-8S group exhibited two degradation stages with corresponding maximum decomposition temperatures of 73.83 °C and 303.73 °C (Figure 5D), attributed to carbon skeleton fragmentation [40]. This indicated that co-assembly behavior enhanced the thermal stability of BSA and 8-Shogaol through a more compact structural composition.

### 3.9. Stability Analysis

**Figure 6:**
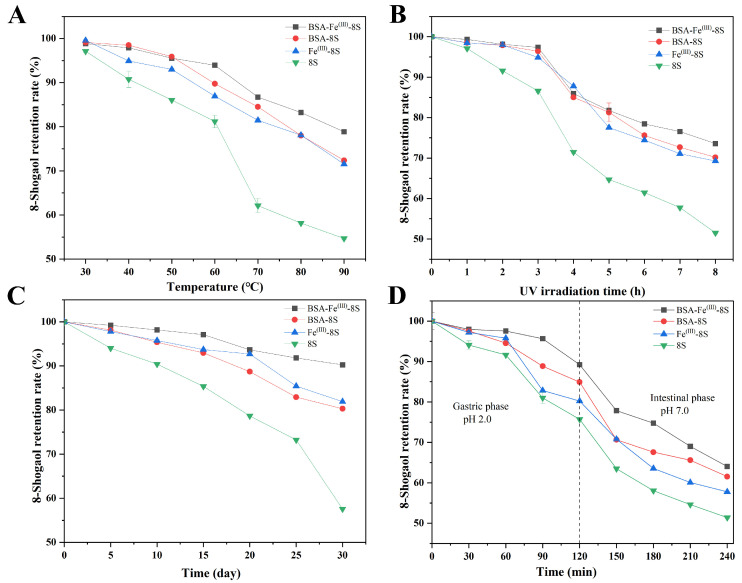
(**A**) Thermal stability, (**B**) UV radiation stability, (**C**) storage stability, (**D**) and in vitro simulated digestion stability of 8-Shogaol, BSA-8S, Fe^(III)^-8S, and BSA-Fe^(III)^-8S NPs. Data are expressed as mean ± standard deviation (*n* = 3) (*p* < 0.05).

Considering the conditions encountered during the preparation, consumption, and transportation of food and pharmaceuticals, a comprehensive stability analysis was conducted on the 8-Shogaol, BSA-Fe^(III)^, Fe^(III)^-8S, and BSA-Fe^(III)^-8S nanoparticles to evaluate their potential for practical applications. Figure 6A presented the retention rates of 8-Shogaol, BSA-Fe^(III)^, Fe^(III)^-8S, and BSA-Fe^(III)^-8S at different temperatures. The release rate of the 8-Shogaol monomer gradually increased with rising temperature, reaching a retention rate of 54.70% at 90 °C. Compared to the 8-Shogaol monomer, the 8-Shogaol release from BSA-Fe^(III)^, Fe^(III)^-8S, and BSA-Fe^(III)^-8S nanoparticles gradually increased with rising temperature but remained significantly lower than the 8-Shogaol group. Their retention rates at 90 °C were 71.54%, 72.38%, and 78.86%, respectively. The results showed that the BSA-Fe^(III)^-8S group exhibited the strongest thermal stability. Fe^(III)^, acting as a cross-linking agent, formed robust bridges between BSA and 8-Shogaol through coordinate bonds, constructing a metal–phenol–aldehyde network.

As observed in Figure 5, the thermal decomposition temperature of the BSA-Fe^(III)^-8S ternary nanoparticle composite was significantly higher than that of pure BSA, indicating that the composite formation substantially enhanced the thermal stability of BSA. Similar results were reported by Ma et al. [23], where ZLC NPs exhibited optimal thermal stability at 90 °C, retaining 95.31% of their structure, compared to 81.65% retention for the CGA monomer. Figure 6B shows the retention rate changes of 8-Shogaol, BSA-Fe^(III)^, Fe^(III)^-8S, and BSA-Fe^(III)^-8S nanoparticles under different UV irradiation durations (0–8 h). As UV exposure time increased, varying degrees of release occurred in the 8-Shogaol content of all groups. The 8-Shogaol monomer group exhibited the poorest UV resistance, with a retention rate of 71.47% after 4 h. The retention rates for the BSA-Fe^(III)^, Fe^(III)^-8S, and BSA-Fe^(III)^-8S nanoparticles were 85.02%, 87.79%, and 85.94%, respectively. After 8 h of UV irradiation, the retention rate of the 8-Shogaol monomer group was 51.50%, while the retention rates for the BSA-Fe^(III)^, Fe^(III)^-8S, and BSA-Fe^(III)^-8S nanoparticle groups were 69.27%, 70.18%, and 73.57%, respectively. UV irradiation disrupts the native structure of proteins, leading to molecular rearrangement that affects BSA aggregation [41]. The results indicated that the BSA-Fe^(III)^-8S ternary nanoparticles exhibited the strongest resistance to UV irradiation and the highest stability.

Storage stability is a key indicator for assessing nanoparticle stability. As storage time increased (0–30 days), the retention rate of the 8-Shogaol monomer group decreased significantly to 57.56% (30 days). Compared to the 8-Shogaol monomer, the BSA-Fe^(III)^, Fe^(III)^-8S, and BSA-Fe^(III)^-8S nanoparticles exhibited slow release of 8-Shogaol with increasing temperature, achieving retention rates of 80.30%, 81.92%, and 90.24% at 30 days. BSA-Fe^(III)^-8S demonstrated exceptionally high storage stability.

### 3.10. In Vitro Digestion Simulation of 8-Shogaol

8-Shogaol is highly unstable and prone to degradation. To enhance its functionality within the body, it is necessary to improve its chemical stability and slow-release properties during gastrointestinal digestion. In vitro simulation of gastrointestinal digestion for 8-Shogaol, BSA-Fe^(III)^, Fe^(III)^-8S, and BSA-Fe^(III)^-8S nanoparticles is shown in Figure 6D. At the end of the gastric digestion phase, the retention rates for the 8-Shogaol, BSA-Fe^(III)^, Fe^(III)^-8S, and BSA-Fe^(III)^-8S groups were 75.69%, 80.23%, 84.92%, and 89.22%, respectively. The retention rate of the nanoparticle groups was significantly higher than that of the 8-Shogaol monomer group, particularly the BSA-Fe^(III)^-8S group, indicating that co-assembly behavior enhanced the stability of 8-Shogaol. On the other hand, phenolic compounds exhibited high stability during simulated gastric digestion. Chlorogenic acid/zein composite nanoparticles also released chlorogenic acid during gastric digestion, consistent with the results of this experiment [23]. During intestinal digestion, the concentrations of 8-Shogaol, BSA-Fe^(III)^, Fe^(III)^-8S, and BSA-Fe^(III)^-8S groups decreased significantly. The 8-Shogaol monomer group exhibited the lowest concentration (51.38%), while the BSA-Fe^(III)^-8S group showed the highest (64.04%). This phenomenon is likely attributed to the fact that Fe^(III)^ chelation and BSA encapsulation effectively shield 8-Shogaol from the alkaline environment of the intestinal phase, where free phenolic compounds are prone to oxidation and degradation. This further demonstrated that Fe^(III)^ chelation and BSA coating enhanced the stability and slow-release properties of 8-Shogaol, establishing a theoretical basis for improving its bioaccessibility.

### 3.11. Cell Viability

**Figure 7:**
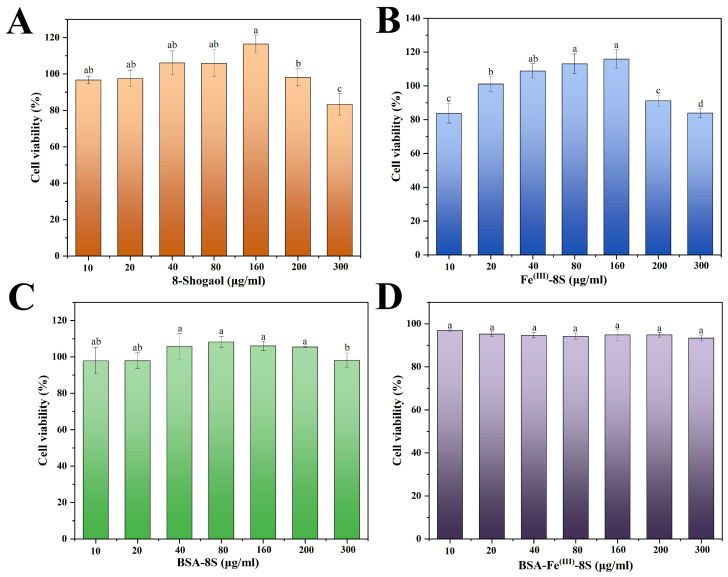
Cytotoxicity in (**A**) 8-Shogaol, (**B**) Fe^(III)^-8S, (**C**) BSA-8S, and (**D**) BSA-Fe^(III)^-8S NPs. Data are expressed as mean ± standard deviation (*n* = 3) (*p* < 0.05). Different lowercase letters indicate significant differences among the groups (*p* < 0.05).

It is well established that 8-Shogaol, the principal bioactive constituent of the edible medicinal plant ginger, exhibits robust anti-inflammatory and antitumor activities. The in vitro cytotoxicity of 8-Shogaol, BSA-Fe^(III)^, Fe^(III)^-8S, and BSA-Fe^(III)^-8S was evaluated via the MTT toxicity assay. Figure 7 showed that all samples at different concentrations exhibited no cytotoxicity, with cell survival rates exceeding 80%. Notably, the 8-Shogaol monomer group, BSA-Fe^(III)^ group, and Fe^(III)^-8S group demonstrated low-concentration cell growth promotion, potentially related to the physiological activities of 8-Shogaol and Fe^(III)^. Fe^(III)^ is an essential nutrient critical for human physiology, while BSA is a biocompatible, metabolizable protein with inherent physiological compatibility. These attributes collectively confer an excellent safety profile on the BSA-Fe^(III)^-8S nanoparticle system, positioning it as a promising platform for therapeutic translation [42,43,44].

## 4. Conclusions

In this study, a novel self-assembled BSA-Fe^(III)^-8S metal–phenolic network nanoparticle was successfully developed. Through condition optimization, the resulting nanoparticles exhibited a uniform spherical morphology with a particle size of 115.13 nm, zeta potential of +52.23 mV, and high encapsulation efficiency (94.93%) and loading capacity (23.73%). Fluorescence quenching analysis confirmed that 8-Shogaol interacts with BSA and BSA-Fe^(III)^ via a static quenching mechanism, with the latter showing stronger binding affinity. The BSA-Fe^(III)^-8S nanoparticles demonstrated superior stability against thermal, UV, and storage stresses compared to free 8-Shogaol and binary control groups. Furthermore, the nanoparticles effectively protected 8-Shogaol during simulated gastrointestinal digestion, achieving a higher retention rate than the free compound. Cytotoxicity evaluation confirmed the biocompatibility of the system. Collectively, these findings highlight the potential of the BSA-Fe^(III)^-8S ternary system as an effective delivery platform for stabilizing bioactive but labile phenolic compounds. Its favorable physicochemical properties and enhanced stability make it a promising candidate for incorporation into functional foods, beverages, and nutraceutical formulations, where protection of sensitive ingredients during processing, storage, and gastrointestinal transit is critical.

## Figures and Tables

**Table 1:** Encapsulation efficiency and loading capacity in different nanoparticle groups.

Samples	EE (%)	LC (%)
Fe^(III)^-8S	84.54 ± 0.04 ^b^	21.14 ± 0.01 ^b^
BSA-8S	84.77 ± 0.00 ^b^	21.20 ± 0.00 ^b^
BSA-Fe^(III)^-8S	94.93 ± 0.04 ^a^	23.73 ± 0.01 ^a^

The superscript letters a and b in the same column indicated significant differences estimated by Duncan’s multiple range test (*p* < 0.05). Different lowercase letters indicate significant differences among the groups (*p* < 0.05).

**Table 2:** Parameters calculated by Stern–Volmer equation and the double-logarithmic curve equation.

Samples	K_sv_ (10^3^ mol^−1^ L)	K_q_ (10^11^ mol^−1^ L S^−1^)	K_a_ (mol^−1^ L)	n
BSA-8S	1.7850 ± 0.0075	5.9500 ± 0.2484	50,644 ± 292.8460	1.0937 ± 0.0447
BSA-Fe^(III)^-8S	2.1720 ± 0.0006	7.2400 ± 0.2011	8,710 ± 331.3146	0.9197 ± 0.3036

## Data Availability

The original contributions presented in this study are included in the article and in supplementary matterials. Further inquiries can be directed at the corresponding author.

## Supplementary Materials

The following supporting information can be downloaded at: https://www.mdpi.com/article/10.3390/foods15081365/s1. Figure S1: The chemical structure of 8-Shogaol.

## Abbreviations

The following abbreviations are used in this manuscript:

8S	8-Shogaol
BSA	Bovine serum albumin
PDI	Polydispersity index
FTIR	Fourier transform infrared spectroscopy
AFM	Atomic force microscopy
TEM	Transmission electron microscopy
TGA	Thermal gravitational analysis
UV–vis	Ultraviolet–visible spectra
XPS	X-ray photoelectron spectroscopy
