# Supplementary figures and images for: BSA-Coated Metal–Phenolic Complex Assembly of 8-Shogaol Nanoparticles: Characterization, Stability, and Slow-Release Properties
Source: Foods. 2026 Apr 14;15(8):1365. doi: 10.3390/foods15081365 (PMC13114892; doi:10.3390/foods15081365)

## Supplementary Materials

Figure S1. The chemical structure of 8-Shogaol

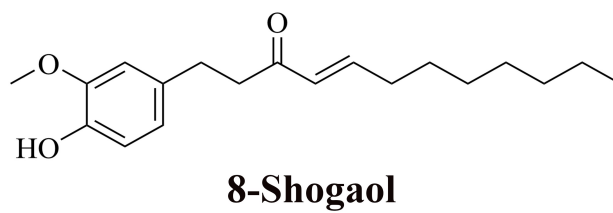

Supplement: Supplementary file 1 [file foods-15-01365-s001.zip › Figure S1.pdf]
